# High Virulence of African Swine Fever Virus Caucasus Isolate in European Wild Boars of All Ages

**DOI:** 10.3201/eid1804.111813

**Published:** 2012-04

**Authors:** Sandra Blome, Claudia Gabriel, Klaas Dietze, Angele Breithaupt, Martin Beer

**Affiliations:** Friedrich-Loeffler-Institut, Greifswald–Insel Riems, Germany (S. Blome, C. Gabriel, A. Breithaupt, M. Beer);; Food and Agriculture Organization of the United Nations, Rome, Italy (K. Dietze)

**Keywords:** African swine fever virus, African swine fever, African swine fever virus Caucasus isolate, European wild boar, adult boars, viruses, Europe

**To the Editor:** African swine fever (ASF) is a serious disease that is currently affecting domestic pigs and wild boars in the Russian Federation. The disease is caused by *African swine fever virus* (ASFV; family *Asfarviridae*), and its continuing spread imposes a growing risk for introduction to disease-free areas with a high density of pigs and/or wild boars. We recently reported on the experimental characterization of ASFV Caucasus isolates in European wild boar piglets and juveniles (*1*), age classes that were deemed to be the most susceptible to ASFV. The extreme virulence of the virus strain led to an almost peracute disease and 100% mortality. On the basis of these data, a scenario of endemicity driven by chronically diseased animals or ASFV carriers seems unlikely. Nevertheless, ASF continues to occur in wild boars.

The clinical course of some infectious diseases is age dependent; thus, we supplemented our previous study (*1*) with a limited study among adult wild boars to help clarify their role in the epidemiology of ASFV. To achieve this goal, we orally inoculated 1 boar (10 years of age), 2 sows (4 and 5 years, respectively), and 1 boar piglet with a 3 × 10^6^ 50% tissue culture infectious dose of the ASFV Caucasus isolate.

Severe, unspecific clinical signs (fever, depression, anorexia, dyspnea, ataxia) developed in all animals. Infection was confirmed by PCR of blood samples and fecal and oral swab samples obtained 6 days after inoculation. All animals died or were euthanized in a moribund state 8–9 days after inoculation, confirming that ASFV causes severe, acute disease and is fatal for 100% of infected adult European wild boars. No antibodies were detected in serum samples throughout the experiment.

The available data show no indication of chronic ASF disease or ASFV carrier states among adult wild boars, conditions that could potentially contribute to long-term persistence of disease in an affected region. In terms of risk assessment, the most likely routes for the introduction of ASFV into wild boar populations are spillover from domestic pigs, exposure to ASFV-contaminated carcasses under climate conditions favoring the persistence of infectious virus, contact with fomites, and consumption of ASFV-contaminated animal feed.
